# Revisiting the determinants of CO2 emissions: The role of higher education under the extended STIRPAT model

**DOI:** 10.1371/journal.pone.0319930

**Published:** 2025-03-18

**Authors:** Qiang Li

**Affiliations:** School of Humanities and Social Sciences, North China Electric Power University, Beijing, China; University of Kalyani, INDIA

## Abstract

This study directly aligns with Sustainable Development Goals (SDGs), i.e., SDG-13 and SDG-4. Carbon emissions (CO2e) are primarily addressed under SDG-13: Climate Action, which aims to combat climate change and its impacts. CO2e reduction efforts contribute to achieving this goal by mitigating greenhouse gas emissions. SDG 4: Quality Education aims to ensure inclusive and equitable quality education for all. It emphasizes explicitly lifelong learning opportunities and targets higher education (HE) access to improve skills for sustainable development. Therefore, the current study aims to examine the determinants of CO2e in China and the role of HE under the extended STIRPAT model. This study utilizes the Fully Modified Ordinary Least Squares (FMOLS) and Dynamic Ordinary Least Squares (DOLS) methods using the time series data from 1985 to 2023. The finding shows that total population, GDP, and industry positively affect CO2e, while technological innovation and higher education negatively affect CO2e in China.

## 1. Introduction

One of the biggest problems facing the globe today is climate change, which is thought to be caused mainly by carbon dioxide emissions (CO2e). CO2e has risen sharply in recent decades due to the rise in energy consumption brought on by population growth and industrialization. Nonetheless, the United Nations and other international leaders are acting seriously to reduce CO2e emissions into the environment [1]. CO2e affects global warming and climate patterns, among other things. Due to its catastrophic effects on several critical sectors, including agriculture, water supplies, human health, population migration, and the loss of several species [1,2]. However, in addition to these severe problems brought on by CO2e, human activities like burning fossil fuels, deforestation, and industrial processes have increased dramatically in recent years, raising the atmospheric concentration of CO2e and raising worries about climate change and global warming. The growing need for energy for industry, transportation, and other uses is one of the primary causes of the rise in carbon emissions. However, it is anticipated that the capacity for renewable energy sources will grow by 50% globally between 2019 and 2024, potentially reducing the adverse impacts of greenhouse gas emissions on the environment and human health [3].

The growth of society, whether it be in its social, economic, political, or moral facets, is significantly influenced by education (EDU). The 17 Sustainable Development Goals (SDGs) that the United Nations (UN) adopted in 2015 addressed various issues, such as environmental, technical, and socioeconomic development. All people should have equitable access to postsecondary education, especially at institutions that support possibilities for lifelong learning, according to SDG Goal 4 [4]. Education may improve the quality of the environment by increasing environmental consciousness, creating energy-efficient and green technology, and producing renewable energy [5,6]. However, education depending on the nation’s economic development levels, education can harm environmental quality by promoting economic growth through human capital, innovation, competitiveness, and entrepreneurship. Governments may also utilize education to fight environmental deterioration [6]. By reducing CO2 emissions, human capital, especially action, contributes significantly to improving environmental sustainability. According to the literature, education contributes to sustainable growth and environment. Some of the most recent research offers this proof; for instance [7], showed a negative correlation between CO2 emissions and education. In a similar way [8], found that while a decrease in educational attainment has increased CO2 emissions in BRICS nations, an increase in education considerably reduces CO2 emissions. Zafar et al. [9] found a strong correlation between environmental deprivation and education. According to Xin et al. [10], human capital factors like the average year of education and the literacy rate reduce CO2 emissions over time for the Chinese economy.

In the literature there are many social, energy, institutional, political, economic, demographic, technological, and health etc determinants of CO2e. Bhattacharya et al. [11] focused on, renewable energy and institutions factors on CO2e, stock market growth, FDI and renewable energy by Paramati et al. [12] and Cai et al. [13], renewable and non-renewable energy consumption and real income by Dogan & Ozturk [14], population growth and economic growth by Sulaiman and Abdul-Rahim [15], economic growth, renewable energy, energy consumption, financial developments, trade openness, and urbanization by Khoshnevis Yazdi and Ghorchi Beygi [16], renewable energy consumption, agriculture production and forest by Waheed et al. [17], agricultural productivity by Eyuboglu and Uzar [18] and Zhou et al. [19], human capital, exports, and economic growth by Rahman et al. [20], education expenditure, and female employers by Uz-Zaman et al. [21], industrialization by Mentel et al. [22], socioeconomic factors by Zhou et al. [23] and Yin et al. [24], secondary industries, and, urbanization level, by Zheng et al. [25], arable land, financial development, globalization, urbanization, and innovation by Azam et al. [26], Industrialization, and Natural Resources by Voumik et al. [27], energy productivity, economic policy uncertainty, institutional quality and geopolitical risk by Cui et al. [28], energy productivity, technological development, and human capital by [29]. Based on the above-mentioned, renewable energy, technology, energy productivity, financial development, and institutional quality positively affect environmental sustainability. In contrast, non-renewable energy, GDP, urbanization, and population all have negative effects on environmental sustainability.

The Chinese country chose this study for several reasons, one of which is that they are passionate about economic growth. According to the World Resources Institute’s CAIT database, China is the world’s greatest yearly emitter of greenhouse gases, accounting for 27% of worldwide emissions in 2020 with 12.3 billion tonnes of CO2 equivalent [30]. Since China’s human CO2e make up over 30% of global emissions, reducing its emissions is essential to mitigating climate change worldwide. The Chinese government has promised to cut CO2 emissions by 60–65% from 2005 levels by 2030 when they are at their highest per GDP unit. To meet the short-term carbon peak target, it is now crucial to analyze how economic policies and other variables affect CO2 emissions as well as anticipate CO2 emissions in order to create CO2 emission strategies and standards [31].

The central objective of this study is to analyze the determinants of CO2e with a special focus on higher education. To the best of the authors’ knowledge, no study has investigated the collection of explanatory variables in the context of China’s economy as investigated by this study. Thus, this study’s contribution is threefold: firstly, unlike the previous studies, this study simultaneously examined the effects of total population, gross domestic product, industry, technological innovation, and higher education in China with a broader aim to achieve sustainable development goals. Secondly, this study model builds on the extended STIRPAT model by adding the higher education variable. Finally, the outcome of this research provides valuable policy recommendations to raise environmental sustainability in the Chinese economy.

The remainder of this article is organized as follows: Section 2 presents prior theoretical and empirical reviews. Section 3 deals with the data and empirical methodology. Section 4 interprets the results. Finally, section 5 deals with the study’s conclusions.

## 2. Literature review

### 2.1. Determinants of CO2 emissions

Zheng et al. [25] examined the determinants of CO2e in 73 Cities in China. They discover a nonlinear pattern in the correlation between CO2e and GDP. This study extends the STIRPAT model by using a linear mixed effect model that covers the quadratic term of GDP per capita to characterize such multimodality and investigate the factors influencing CO2 emissions in these cities. The findings showed that CO2e in Chinese cities has typically been positively impacted by factors such as population size, the percentage of secondary industries, energy consumption structure, urbanization level, and economic level. Zhou et al. [23] analyzed the socioeconomic determinants of CO2e in China using time series data from 1980–2014 using the VECM methods. They found that socioeconomic factors such as energy consumption, income, urbanization, FDI, and total trade significantly impacted CO2e. Yin et al. [24] examined the asymmetric socioeconomic determinants of CO2e in China using the nonlinear ARDL technique from 1980–2019. The findings indicated that while a decline in economic growth balances CO2e in China, a positive shift in economic growth is the primary driver of CO2e rise. Concurrently, both positive and negative changes in energy consumption have long-term adverse effects on CO2e, but negative shocks have less effect on CO2e than positive energy shocks. In the long term, shocks and positive school years are helpful in China’s fight against CO2e. Azam et al. [26] examined the impact of urbanization, globalization, and energy consumption on CO2e in the SAARC region under the extended STIRPAT model. By utilizing the CS–ARDL methods from 1990–2018. They found that CO2e are caused by urbanization, GDP per capita income, energy consumption, industrial expansion, globalization, and financial development, while other variables, such as innovation and arable land, negatively impact them. Voumik et al. [27] examined the association between urbanization, industrialization, natural resources rent, and Anthropogenic CO2e in South Asia from 1972 to 2021 by using the CS-ARDL Approach. They modelled the variable in the STIRPAT. They found that industrialization, urbanization, and economic growth raise CO2e. Electrification, natural resource rent, and population growth contributed to lower CO2e. Uddin et al. [32] examined the impact of geopolitical risk, governance, technological innovations, energy use, and foreign direct investment on CO2e in the BRICS region from 1990 to 2018 by using the CS ARDL. They found that government effectiveness, regulatory quality, the rule of law, foreign direct investment (FDI), and innovation have a negative effect on CO2e. In contrast, geopolitical risk, corruption, political stability, and energy consumption have a positive effect on CO2e. Du et al. [29] used data from 1990 to 2018 to examine the Environmental Kuznets Curve (EKC) hypothesis, focusing on how energy productivity, technological development, and human capital contribute to a sustainable environment in 35 OECD economies. The study supports an N-shaped EKC hypothesis link between GDP and CO2e. They found that energy productivity, technological development, and human capital negatively affect CO2e. Using the CS-ARDL, Cui et al. [28] investigated the effects of energy productivity, institutional quality, economic policy uncertainty, and geopolitical risk in BRICS from 1992 to 2021. The study finds that FDI, energy productivity, and institutional quality have positive effect on CO2e.

### 2.2. Education and CO2 emissions nexus

Misra and Verma [33] examined environmental education and CO2e mitigation. They found that educational initiatives assist in lowering anthropogenic CO2e, increasing the execution rate of educational programs and the rate at which education is disseminated among people cannot effectively reduce CO2e if the rate at which educated individuals reduce their carbon footprint is low. Alkhateeb et al. [34] using data from 1971 to 2014, to examined economic development, energy use, education and CO2e nexus in Saudi Arabia. They discovered that secondary education may have a negative impact on CO2e, but primary education does not. Whereas energy consumption raise CO2e, it also confirmed that an inverted U-shaped ECK exists. Eyuboglu and Uzar [35] examined the association between higher education and CO2e in Turkey by utilizing the ARDL and VECM methods. The findings show that CO2e is adversely impacted by higher education. Energy use and economic expansion also have both short-term and long-term favorable effects on CO2e. Li and Ullah [8] used a nonlinear panel ARDL technique to investigate the relationship between human capital and CO2e in BRICS from 1991 to 2019. The results indicated that an adverse change in education has eventually led to higher CO2e, and a positive change in education has decreased CO2e. Using the EKC framework, Lee et al. [36] examined the territory education and CO2e nexus in 151 countries from 1991 to 2019. The results support the EKC and imply that in nations with higher GDP per capita, tertiary education helps to reduce CO2e. Sahu et al. [37] examined the relationships between urbanization, industrialization, FDI, and CO2e in 31 nations. It also examines the moderating effects of education on these associations from 1998 to 2020 by using the GMM methods. They showed that urbanization and industry raise CO2e levels, while industry and education have a negative impact on CO2e. Khan et al. [4] analyzed the impact of women’s education on CO2e in Pakistan from 1995–2021 using ARDL methods. They found that women’s education, education spending, technological innovation and renewable energy contribute to lower CO2e.

### 2.3. Education and CO2 emissions Nexus in China

Li et al. [38] analyzed the HE and CO2e nexus evidence from 30 provinces in China from 2000–2018 using the FMOLS and DOLS estimators. The findings supported the pollution halo theory and the education-CO2-driven hypothesis by demonstrating the critical roles of FDI and higher education in reducing CO2e. At the same time, CO2e emissions were significantly influenced by increases in population, gross domestic product, and electricity use. Zhu et al. [39] analyzed the relationship between regional CO2e and China’s higher education system using data from 31 Chinese provinces between 2004 and 2015 using the panel threshold regression and panel quantile regression models. The findings show that the extent and quality of higher education in China have a threshold influence on regional CO2e. More specifically, when technology advances beyond a certain point, more education may benefit CO2e. Regarding the quality of higher education, its continued expansion may mitigate the beneficial impact on CO2e when income surpasses a certain threshold; it may also exacerbate its limited impact on carbon emissions per capita when technology surpasses a certain threshold; and ongoing enhancement of higher education quality may contribute to a decrease in CO2e. Uz-Zaman et al. [21] analyzed the association between EDU expenditure and CO2e in China from 1991 to 2015 by utilizing the ARDL technique. The results showed that raising education spending, hiring more women as employers, and using more renewable energy as a proportion of total energy usage will all contribute to China’s long- and short-term CO2 emission reduction. Liu et al. [7] analyzed the association between financial inclusion, EDU and CO2e nexus in China by using the ARDL approach. They found that education research and development activities and four of the five financial inclusion proxies positively affect China’s environmental quality. Meanwhile, GDP and population had a negative effect on CO2e.

Cui et al. [40] assessed how different educational levels affect China’s CO2e under the EKC. The Primary, secondary, and tertiary enrollment, as well as the average year of education, were among the disaggregated and aggregated data used in the research. In the long term, all of the proxies of education positively influence CO2e; however, the impact of education on CO2 emissions turns negative when we consider the square of these variables. The study also supported the EKC hypothesis. Xin et al. [10] worked on unemployment, EDU and CO2e nexus by utilizing data from the Chinese economy covering the years 1991–2020 by utilizing the ARDL methods. The vibrant side of human capital demonstrates how factors like the average year of education and the literacy rate reduce CO2 emissions over time. Furthermore, the volume and direction of human capital results are also grounded on facts. Additionally, empirical research has shown that, over time, unemployment considerably raises CO2e. Lin et al. [41] examined the education level of residents ‘ CO2e in 30 Chinese provinces. They found that the influence of educational attainment, the average yearly efficiency and the average efficiency of each indicator have grown in most provinces. Second, the efficiency of carbon emission indicators in both urban and rural regions is highest in the East, followed by the West, and lowest in the centre region, both before and after taking exogenous variables into account. The third finding is that the efficiency of carbon emission indicators in cities is higher in the East than in rural areas and lower in the middle and Western regions.

Dong et al. [42] utilized panel data from 266 Chinese cities spanning the period from 2009 to 2020 to analyze the impact of education investment on carbon dioxide emissions by using the both static and dynamic spatial Durbin model regressions are employed. The findings indicated that education investment plays a significant role in reducing carbon dioxide emissions, both through spatial spillover effects and dynamic effects over time. When education investment is implemented in one city, it positively influences neighboring cities, contributing to a measurable reduction in carbon emissions across the region. Zhao et al. [43] examined the county-level data from China spanning 2007–2021 to estimate the impact of local government education expenditure on air pollution. The results reveal that education expenditure has a significant negative effect on air pollution, a finding that remains robust after accounting for endogeneity. Mechanism analysis shows that education expenditure mitigates air pollution through composition effects, technique effects, and income effects.

### 2.4. Research gap

Based on the above literature, some researchers, such as Zheng et al. [25], analyzed the determinants of CO2e in 73 Chinese cities, Azam et al. [26], examined the impact of urbanization, globalization, and energy consumption on CO2e in the SAARC region, and Voumik et al. [27], investigated the relationship between urbanization, industrialization, natural resource rent, and CO2e in South Asia, used the extended STIRPAT model. However, they overlooked the inclusion of higher education in the model. The relationship between education and CO2 emissions in China has been explored by Li et al. [38], Zhu et al. [39], Uz-Zaman et al. [21], Liu et al. [7], Cui et al. [40], Xin et al. [23], Lin et al. [41], Dong et al. [42], and Zhao et al. [43], but none of these studies employed the STIRPAT model. According to the available literature, no studies have specifically examined the factors affecting CO2e in China with a focus on higher education under the extended STIRPAT model. Therefore, this study aims to fill the gap by investigating the determinants of CO2e in China, incorporating the role of higher education within the STIRPAT framework.

### 2.5. Theoretical review

Eyuboglu and Uzar [35] developed the theoretical framework for higher education (HE) to promote environmental sustainability. HE influences human capital, income distribution, and environmental quality. Higher education builds human capital through skills and knowledge, which support environmental technologies that improve environmental quality. Courses, workshops, and conferences promote environmental awareness, increasing the demand for environmental quality and encouraging sustainable practices. Fair income distribution helps create a balanced society, indirectly benefiting the environment. Fixed capital and infrastructure investments drive economic growth but can harm the environment if not managed sustainably. Energy consumption, if excessive, negatively impacts environmental quality, highlighting the need for responsible resource use. These interconnected factors illustrate the role of education and equitable growth in enhancing environmental sustainability [35]. EDU can be crucial for raising environmental awareness and developing human capital [44]. Environmental awareness may also be increased by offering environmental courses, seminars, conferences, and workshops at schools and higher education institutions [45].

## 3. Methodology and data

### 3.1. Empirical model

This study uses an extended version of the STIRPAT model to examine the determinants of CO2e in china with special focused on higher education. The STIRPAT model, proposed by Dietz and Rosa [46], is a mathematical extension of the IPAT model. Originally introduced by Ehrlich and Holdren [47], the IPAT model represents environmental (I) impact as a product of three factors: population (P), affluence (A), and technology (T), expressed in Equation (1):


I=P×A×T (1)

Despite its usefulness, the IPAT model has been criticized for its assumption of fixed proportionality among variables, limiting its ability for hypothesis testing [48]. To address these limitations, Dietz and Rosa [46], introduced the STIRPAT model, which generalizes IPAT to allow more flexible relationships between variables. The general form of the STIRPAT model is:


$$I_{t}=\alpha P_{t}^{\delta_{1}}A_{t}^{\delta_{2}}T_{t}^{\delta_{3}}e_{t}$$ (2)

According to Anser et al. [49], and Azam et al. [26], a key advantage of the STIRPAT model is the flexibility to add additional variables based on the objective of the study. This study extends Equation (2) by incorporating higher education (HE) as an additional factor:


$$I_{t}=\alpha P_{t}^{\delta_{1}}A_{t}^{\delta_{2}}T_{t}^{\delta_{3}}HE_{t}^{\delta_{4}}e_{t}$$ (3)

Many studies transform Equation (3) into its logarithmic form to achieve a linear relationship. The logarithmic form is given in Equation (4):


$$LnI_{t}=\alpha +\delta_{1}lnP_{t}+\delta_{2}lnA_{t}+\delta_{3}lnT_{t}+\delta_{4}lnHE_{t}+e_{t}$$ (4)

CO2e, and total population, use as a proxy for *I* and *P*, GDP and industry use as proxy for *A*, while technological innovation use as proxy for *T* [26]. To examine the impact of socio-economic indicators on CO2e in china, the specific STIRPAT model is specified as:


$$\text{CO}2{\text{e}}_{\text{t}}=\alpha +\delta_{1}{\text{TP}}_{\text{t}}+\delta_{2}{\text{GDP}}_{\text{t}}+\delta_{3}{\text{IND}}_{\text{t}}+\delta_{4}{\text{TI}}_{\text{t}}+\delta_{4}{\text{HE}}_{\text{t}}+{\text{e}}_{\text{t}}$$ (5)

In Equation (5), CO2e, TP, GDP, IND, TI and HE denote the CO2 emissions, total population, gross domestic product, industry, technological innovation, and higher education respectively. Where *t* represents a time series from (1985 to 2023), CO2e is dependent variable, and independent variables are TP, GDP, IND, TI and HE.

### 3.2. Estimation technique

In this study, we initially perform unit root tests, including the Augmented Dickey-Fuller (ADF) test and the Phillips-Perron (PP) test. The ADF test, introduced by Dickey and Fuller [50], and the PP test, developed by Phillips and Perron [51], are used to determine the stationarity of time series data. The ADF test is formulated in Equation (6):


$$\text{Δ}G_{t}=\emptyset +\omega t+\delta G_{t-1}+\sum_{j=1}^{q}\vartheta_{j}\text{Δ}G_{t-j}+e_{t}$$ (6)

where $\text{Δ}G_{t}$ represent the first difference of the series,  ∅  is a drift term, ωt denotes a deterministic time trend, $\delta G_{t-1}$ is the lagged level of the series, and $\sum_{j=1}^{q}\vartheta_{j}\text{Δ}G_{t-j}$ accounts for the lagged differences of the dependent variable to correct for autocorrelation. The term $e_{t}$ represents a white noise error term, while *q* is the number of lagged difference terms included in the model. The null hypothesis $\left(H_{0}\right)$ for this test states that δ=0, indicating the presence of a unit root and thus non-stationarity in the series. Conversely, the alternative hypothesis $\left(H_{1}\right)$ posits that δ<0, suggesting that the series is stationary. The PP test differs from the ADF test in that it employs non-parametric methods to adjust the *t* statistic of the *δ* coefficient, correcting for serial correlation and heteroskedasticity in the residuals without requiring the inclusion of lagged difference terms [52].

After the unit root test, we will use the Cointegation technique such as (JJ) Johansen and Juselius [53], Cointegation. The JJ approach is ideal for multivariate time series to detect long-run equilibrium relationships. After the Cointegration, we thirdly employs the fully modified OLS (FMOLS) and dynamic OLS (DOLS) for the long run estimates. Phillips and Hansen (1990) developed the FMOLS to apply an ideal co-integrating regression estimate. However, because it offers the benefit of reducing endogeneity bias and serial correlation, the Pedroni [54] heterogeneous FMOLS estimator was utilized for the panel cointegration regression. Considering that a panel FMOLS estimator is used for the coefficient in Equation (7):


$$\propto_{NT}^{\text{*}}-\propto =\left(\sum_{i=1}^{N}L_{22i}^{-2}\sum_{i=1}^{T}{\left(Y_{i}-Y_{i}\right)}^{2}\right)\;\sum_{i=1}^{N}L_{11i}^{-1}L_{22i}^{-1}\left(\sum_{i=1}^{T}\left(\chi_{i}-\chi_{i}\right)\mu_{i}^{\text{*}}-T{\hat{\gamma }}_{i}\right),$$ (7)

where,


$$\mu_{i}^{\text{*}}=\mu_{i}-\frac{{\hat{L}}_{21i}}{{\hat{L}}_{22i}}\text{Δ}\chi_{i^{\prime }}{\hat{\gamma }}_{i}={\hat{F}}_{21i}{\overset{\text{ˆ}}{\text{Λ}}}_{21i}^{0}-\frac{{\hat{L}}_{21i}}{{\hat{L}}_{22i}}\left({\text{Γ}}^{\wedge }22i+{\overset{\text{ˆ}}{\text{Λ}}}_{22i}^{0}\right),$$

and ${\hat{L}}_{i}$ was the lower triangulation of ${\hat{\lambda }}_{i}$.

The DOLS had the same asymptotic distribution as that of the panel FMOLS estimation derived by [54]. Both the DOLS and FMOLS estimations were performed as shown to confirm the consistency of the outcome (see, [55]). After the long run estimates we employs the Granger causality test.

The Granger causality test is estimated using the following Equations (8) and (9):


$$Y_{\text{t}}=\alpha_{0}+\overset{k}{\underset{j=1}{\sum }}\alpha_{1s}Y_{\text{t}-s}+\overset{m}{\underset{i=1}{\sum }}\alpha_{2\text{i}}Z_{\text{t}-m}+e_{1t}$$ (8)

$$Z_{t}=\beta_{0}+\overset{n}{\underset{j=1}{\sum }}\beta_{1j}Z_{t-j}+\overset{p}{\underset{h=1}{\sum }}\beta_{2h}Y_{t-h}+e_{2t}$$ (9)

**Fig 1:**
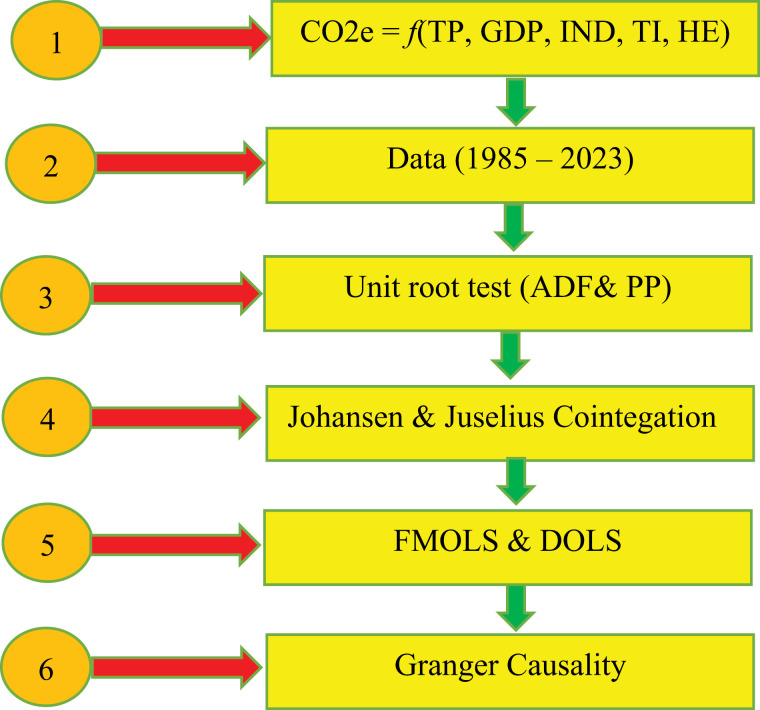
Research framework.

If coefficient of $\alpha_{2i}$ is statistically significant, i.e., $\alpha_{2i}\neq 0$ then Y→ Granger causes →X. The term *Y* and *X* will be independent if $\alpha_{2i}$ and $\beta_{2h}$ are not other than zero. If *X* is cause variable for *Y* and $\beta_{2h}$ is statistically significant, i.e., $\beta_{2h}\neq 0$. The significance of $\alpha_{2i}$ and $\beta_{2h}$ confirms mutual dependency of two specific variables. The term $\varepsilon_{1t}$ and $\varepsilon_{2t}$ are uncorrelated with each other [56,57]. Fig 1, shows the Research framework.

### 3.3. Data and variables

This study examines the determinant of CO2e, with special focused on higher education in china from 1985 to 2023. The data has been obtained from the World Development Indicators (WDI). Table 1, represent the data sources and measurements. The data includes in the Supporting Information see “S1 Table.pdf”.

**Table 1:** Variable measurement.

Symbol(s)	Variable(s)	Measurement(s)	Source(s)
CO2e	Carbon emissions	metric tons per capita)	WDI
TP	Total population	total	WDI
GDP	Gross domestic product	per capita (constant 2015 US$)	WDI
IND	Industry	value added (constant 2015 US$)	WDI
TI	Technological innovation	Total patent application	WDI
HE	Higher education	tertiary (% gross)	WDI

Source: Authors compilation.

## 4. Results and discussion

### 4.1. Descriptive statistics

Table 2, represent the mean value of CO2E, TP, GDP, IND, TI and HE are 8.598, 20.963, 8.040, 27.990, 11.735 and 2.583 respectively. While the Standard deviation of CO2E, TP, GDP, IND, TI and HE are 0.655, 0.088, 0.929, 1.137, 1.925 and 1.172 respectively. The Jarque-Bera statistics all values of statistically insignificance it favors the accepting the null hypothesis of the normality.

**Table 2:** Descriptive statistics.

	CO2E	TP	GDP	IND	TI	HE
Mean	8.598	20.963	8.040	27.990	11.735	2.583
Median	8.615	20.983	8.027	28.062	11.778	2.820
Maximum	9.451	21.069	9.407	29.538	14.277	4.315
Minimum	7.534	20.773	6.503	25.969	8.988	0.785
Std. Dev.	0.655	0.088	0.929	1.137	1.925	1.172
Skewness	-0.090	-0.611	-0.099	-0.265	-0.036	-0.055
Kurtosis	1.457	2.280	1.664	1.761	1.500	1.549
Jarque-Bera	3.923	3.267	2.966	2.951	3.666	3.439
Probability	0.141	0.195	0.227	0.229	0.160	0.179
Observations	39	39	39	39	39	39

### 4.2. Unit root and cointegration test

Table 3 shows the outcomes of the ADF and PP unit root test. At level, The ADF and PP with Constant, with Constant & Trend all variables are non-stationary. After the first difference, the ADF and PP test confirms that all variables becomes stationary. Table 4 shows Cointegration test, both trace and Max-Eigen statistics confirms that 4 Cointegrated equation exist. So therefore we confirms that there is a long run relationship exist between the variables.

**Table 3:** Unit root test.

ADF							
**At Level**							
		CO2E	TP	GDP	IND	TI	HE
With Constant	t-Statistic	-1.249	-1.628	-1.130	-2.202	-0.511	-0.073
	Prob.	0.643	0.459	0.693	0.209	0.878	0.945
With Constant & Trend	t-Statistic	-1.178	0.425	-0.875	-0.480	-1.117	-2.552
	Prob.	0.900	0.999	0.948	0.980	0.913	0.481
**At First Difference**							
With Constant	t-Statistic	-2.820^***^	-4.240^*^	-2.776^***^	-3.730^*^	-5.214^*^	-3.406^**^
	Prob.	0.065	0.000	0.072	0.058	0.000	0.017
With Constant & Trend	t-Statistic	-2.981	-2.578	-2.975	-3.212^**^	-5.204^*^	-3.360^***^
	Prob.	0.151	0.292	0.153	0.098	0.001	0.073
**PP**							
**At Level**							
		CO2E	TP	GDP	IND	TI	HE
With Constant	t-Statistic	-1.188	-0.890	-1.408	-1.171	-0.507	-0.431
	Prob.	0.670	0.781	0.568	0.258	0.879	0.893
With Constant & Trend	t-Statistic	-1.015	-2.178	-0.328	1.142	-1.540	-1.868
	Prob.	0.930	0.488	0.987	1.000	0.798	0.651
**At First Difference**							
With Constant	t-Statistic	-2.902^***^	-4.152^*^	-2.975^**^	-2.250	-5.201^*^	-3.556^**^
	Prob.	0.055	0.000	0.047	0.193	0.000	0.012
With Constant & Trend	t-Statistic	-3.775^**^	-1.523	-3.185	-4.714^*^	-5.204^*^	-3.512^***^
	Prob.	0.027	0.803	0.103	0.000	0.001	0.053

Note:

* ,

** &

*** represent the 1%, 5% & 10% level of significance.

**Table 4:** Cointegration test.

No. of CE(s)	Trace			Max-Eigen	
	Eigenvalue	Statistic	Prob.	Statistic	Prob.
None	0.764	166.610^*^	0.000	53.453^*^	0.001
At most 1	0.662	113.157^*^	0.000	40.091^*^	0.008
At most 2	0.569	73.066^*^	0.000	31.152^*^	0.017
At most 3	0.535	41.914^*^	0.001	28.359^*^	0.004
At most 4	0.284	13.554^***^	0.096	12.376^***^	0.097
At most 5	0.031	1.178	0.278	1.178	0.278

Note:

* ,

** &

*** represent the 1%, 5% & 10% level of significance.

### 4.3. Long run estimates

Table 5, shows the FMOLS and DOLS estimates. In long run the variable total population, GDP, and industry have positive effect on CO2e, while technological innovation, and higher education have negative effect on CO2e in china from 1985 to 2022. The coefficient of TP and GDP is positive, indicating that 1% surge in the total population and GDP leads to rise CO2e by 0.116% and 0.038%. The larger population increases CO2e as more people consume energy, goods, and services, leading to higher fossil fuel usage. This demand drives industrial production, transportation, and energy generation, all of which release CO2e. Economic growth raises CO₂ emissions by boosting industrial production, energy demand, and transportation, all of which often rely on fossil fuels. Increased production and consumption associated with growth lead to higher emissions. Without sustainable practices, economic expansion typically results in a larger carbon footprint. The results consistent with the line of [26,58]. Ochi and Saidi [58] reported that Population and economic growth have a positive and significant influence on climate change in 33 most polluting countries. Azam et al. [26] reported that urban population and GDP have positive effect CO2e in south Asian counties from 1990–2018.

**Table 5:** Long run estimates.

Variable	FMOLS				DOLS			
	Coefficient	Std. Error	t-Statistic	Prob.	Coefficient	Std. Error	t-Statistic	Prob.
TP	0.116^*^	0.038	3.078	0.000	0.451^*^	0.060	7.486	0.000
GDP	0.038	0.507	0.076	0.940	0.324^**^	0.154	2.101	0.047
IND	0.893^*^	0.311	2.874	0.010	0.704^*^	0.031	22.637	0.000
TI	-0.300^**^	0.120	-2.494	0.018	-0.430^***^	0.234	-1.837	0.093
HE	-0.066^*^	0.005	-13.830	0.000	-0.632^***^	0.316	-2.000	0.063
R^2^	0.97				0.99			
Adj. R^2^	0.96				0.98			

Note:

* ,

** &

*** represent the 1%, 5% & 10% level of significance.

The coefficient of IND is 0.893, showing that 1% surge in the industrialization cause to surge CO2e by 0.893%. Industries raise CO₂ emissions through the combustion of fossil fuels for energy in manufacturing, processing, and construction. Industrial processes, like cement and steel production, release significant CO₂ as byproducts. As industrial activity grows, emissions increase unless mitigated by cleaner technologies or energy sources. The finding consistent with the line of Misra and Verma [59], while contradict from [60]. Misra and Verma [59] found that one of the primary causes of the rise in the atmospheric concentration of heat-trapping gases, primarily carbon dioxide (CO2), is the growth of the industrial sector. Industrial CO2 emissions have been a major contributor to global warming and related climatic changes since the beginning of the industrial revolution. Mehmood et al. [60] found that green industrial transformation has negative effect on CO2e.

The coefficient of TI is -0.300, showing that 1% surge in the industrialization cause to reduce CO2e by 0.30%. Thus, Technological innovation reduces CO₂ emissions by improving energy efficiency, enabling cleaner energy sources like wind and solar, and optimizing industrial processes to produce fewer emissions. Innovations such as carbon capture also directly remove CO₂ from the atmosphere. Innovation promotes economic development, productivity gains, and the switch to low-carbon energy sources. Innovation also speeds up technical advancement by permitting increased economic production with the same amount of other inputs. Long et al. [61] reported that environmental innovation positively affects economic growth and environmental quality. Wang et al. [62], Khan et al. [4] and Azam et al. [26] reported that innovation has a favorable effect on environmental quality.

The coefficient of HE is negative and statistical significance sign, indicating that 1% increase in the higher education leads to reduce CO2e by -0.066. Lee et al. (2024) reported that tertiary education helps to reduce CO2e and suggested that Basic social policies like the development of tertiary education can provide as an indirect support for global initiatives aimed at mitigating environmental challenges. Khan et al. [4] reported that women education has fourable effect on environmental sustainability. To ensure the ongoing growth of any nation, both men and women are essential. Increasing women’s literacy and access to high-quality education is one of the best ways to increase female productivity and wellbeing. The achievement of sustainable development is not only made possible by educated women, but also by their enlightened offspring [4,63].

### 4.4. Granger causality analysis

Table 6 shows the Granger causality analyses. The results reported that there is a Uni-directional causality cause from CO2E to TP, GDP to CO2E, IND to CO2E, HE to CO2E, GDP to TP, IND to TP, TP to HE, GDP to TI, GDP to HE, IND to TI, IND to HE and TI to HE. Moreover, Granger causality show that there are bi-directional causality exists between TI and TP.

**Table 6:** Ganger causality test.

Null Hypothesis:	F-Statistic	Prob.	Remarks
TP ⇎ CO2E	1.657	0.207	CO2E ⇒ TP
CO2E ⇎ TP	4.327^*^	0.022	
GDP ⇎ CO2E	5.899^*^	0.000	GDP ⇒ CO2E
CO2E ⇎ GDP	0.661	0.523	
IND ⇎ CO2E	6.139^*^	0.000	IND ⇒ CO2E
CO2E ⇎ IND	0.208	0.813	
TI ⇎ CO2E	1.613	0.215	TI ⇎ CO2E
CO2E ⇎ TI	2.127	0.136	
HE ⇎ CO2E	5.109^*^	0.000	HE ⇒ CO2E
CO2E ⇎ HE	1.617	0.214	
GDP ⇎ TP	4.754^**^	0.016	GDP ⇒ TP
TP ⇎ GDP	2.457	0.102	
IND ⇎ TP	5.447^*^	0.009	IND ⇒ TP
TP ⇎ IND	2.017	0.150	
TI ⇎ TP	2.934^***^	0.068	TI ⇐→ TP
TP ⇎ TI	3.191^***^	0.055	
HE ⇎ TP	2.115	0.137	TP ⇒ HE
TP ⇎ HE	8.018^*^	0.002	
IND ⇎ GDP	1.610	0.216	IND ⇎ GDP
GDP ⇎ IND	0.665	0.521	
TI ⇎ GDP	0.396	0.676	GDP ⇒ TI
GDP ⇎ TI	2.597^***^	0.090	
HE ⇎ GDP	0.171	0.844	GDP ⇒ HE
GDP ⇎ HE	8.737^*^	0.001	
TI ⇎ IND	0.094	0.910	IND ⇒ TI
IND ⇎ TI	3.746^**^	0.035	
HE ⇎ IND	0.034	0.966	IND ⇒ HE
IND ⇎ HE	10.001^*^	0.000	
HE ⇎ TI	1.398	0.262	TI ⇒ HE
TI ⇎ HE	7.961^*^	0.002	

Note:

* ,

** &

*** represent the 1%, 5% & 10% level of significance.

## 5. Conclusion and policy recommendation

This study empirically examined the determinants of CO2e, focusing mainly on higher education in China from 1985 to 2023 using the FMOLS and DOLS estimators. The findings showed that total population, GDP, and industry positively affect CO2e, while technological innovation and higher education negatively affect CO2e in China. This study recommends some suggestions for policymakers to enhance environmental sustainability in China: First, the government should provide funds for research initiatives focused on carbon reduction technologies and practices to drive innovation. Second. Provide incentives for using public transportation, biking, or walking to reduce reliance on fossil fuels. Implement electric vehicle charging stations to encourage the use of low-emission vehicles. Third, to develop a Curriculum that embeds climate change and sustainability topics across all academic programs to foster informed future leaders. Fourth, encourage student participation in sustainability projects to enhance experiential learning. Fifth, facilitate collaborations between universities, government agencies, and private companies to drive research on carbon capture and sustainable practices. Six, establish stringent emission reduction targets and fuel efficiency standards for industries to encourage the adoption of cleaner technologies. This regulatory framework will drive innovation and accountability in reducing CO₂ emissions. Seventh, Promote green growth strategies that decouple economic development from carbon emissions by investing in sustainable industries and technologies. Eight, encourage sustainable urbanization practices focusing on efficient land use, public transportation, and green infrastructure to manage population density and reduce emissions. Ninth, raise public awareness about energy consumption and sustainable practices to encourage lower carbon footprints among individuals and communities. Tenth, support renewable energy, invest in and incentivize the transition to renewable energy sources to meet the growing energy demand without increasing CO₂ emissions.

The limitations of this study include its exclusive focus on China, which restricts the generalizability of the findings to other countries. The study only examined a limited set of determinants of CO2 emissions, specifically higher education, and did not consider other important variables. Additionally, the study will extend to emerging, developing, or developed nations, which could provide a broader perspective. Only FMOLS and DOLS estimators were used, while methods like quintile regression, AMG, and CCEMG estimators were not explored. Furthermore, asymmetric econometric techniques offer an additional avenue for expanding the study’s scope.

## Supporting information

S1 Table S1 Table. (PDF)

## References

[1] Hussain A, Khan F, Albalawi O. Modeling and Monitoring CO2 Emissions in G20 Countries: A Comparative Analysis of Multiple Statistical Models. Sustainability. 2024;16(14):6114. doi: 10.3390/su16146114

[2] Luo M, Qin S, Chang H, Zhang A. Disaggregation Method of Carbon Emission: A Case Study in Wuhan, China. Sustainability. 2019;11(7):2093. doi: 10.3390/su11072093

[3] Jie W, Rabnawaz K. Renewable energy and CO2 emissions in developing and developed nations: a panel estimate approach. Front Environ Sci. 2024;12. doi: 10.3389/fenvs.2024.1405001

[4] Khan F, Uddin I, Don YB, Awan AM. Does women’s education play a role in sustainable environment in Pakistan? A quantitative approach. Environment, Development and Sustainability. 2024;1(1):1–19.

[5] Uddin MMdM. Causal Relationship between Education, Carbon Dioxide (CO2 ) Emission and Economic Growth in Bangladesh. IOSRJHSS. 2014;19(4):60–7. doi: 10.9790/0837-19486067

[6] Sart G, Bayar Y, Danilina M, Sezgin FH. Economic Freedom, Education and CO2 Emissions: A Causality Analysis for EU Member States. Int J Environ Res Public Health. 2022;19(13):8061. doi: 10.3390/ijerph19138061 35805730 PMC9265646

[7] Liu N, Hong C, Sohail MT. Does financial inclusion and education limit CO2 emissions in China? A new perspective. Environ Sci Pollut Res. 2022;1-8. 10.1007/s11356-021-17032-1 34687414

[8] Li X, Ullah S. Caring for the environment: how CO2 emissions respond to human capital in BRICS economies? Environ Sci Pollut Res Int. 2022;29(12):18036–46. doi: 10.1007/s11356-021-17025-0 34677778

[9] Zafar MW, Saleem MM, Destek MA, Caglar AE. The dynamic linkage between remittances, export diversification, education, renewable energy consumption, economic growth, and CO2 emissions in top remittance‐receiving countries. Sustainable Development. 2021;30(1):165–75. doi: 10.1002/sd.2236

[10] Xin Y, Yang S, Faisal Rasheed M. Exploring the impacts of education and unemployment on CO2 emissions. Economic research-Ekonomska istraživanja. 2023;36(2).

[11] Bhattacharya M, Awaworyi Churchill S, Paramati SR. The dynamic impact of renewable energy and institutions on economic output and CO 2 emissions across regions. Renewable Energy. 2017;111:157–67. doi: 10.1016/j.renene.2017.03.102

[12] Paramati SR, Mo D, Gupta R. The effects of stock market growth and renewable energy use on CO2 emissions: Evidence from G20 countries. Energy Economics. 2017;66:360–71. doi: 10.1016/j.eneco.2017.06.025

[13] Cai Y, Sam CY, Chang T. Nexus between clean energy consumption, economic growth and CO2 emissions. Journal of Cleaner Production. 2018;182:1001–11. doi: 10.1016/j.jclepro.2018.02.035

[14] Dogan E, Ozturk I. The influence of renewable and non-renewable energy consumption and real income on CO2 emissions in the USA: evidence from structural break tests. Environ Sci Pollut Res Int. 2017;24(11):10846–54. doi: 10.1007/s11356-017-8786-y 28293824

[15] Sulaiman C, Abdul-Rahim AS. Population Growth and CO2 Emission in Nigeria: A Recursive ARDL Approach. Sage Open. 2018;8(2):2158244018765916. doi: 10.1177/2158244018765916

[16] Khoshnevis Yazdi S, Ghorchi Beygi E. The dynamic impact of renewable energy consumption and financial development on CO2emissions: For selected African countries. Energy Sources, Part B: Economics, Planning, and Policy. 2017;13(1):13–20. doi: 10.1080/15567249.2017.1377319

[17] Waheed R, Chang D, Sarwar S, Chen W. Forest, agriculture, renewable energy, and CO2 emission. Journal of Cleaner Production. 2018;172:4231–8. doi: 10.1016/j.jclepro.2017.10.287

[18] Eyuboglu K, Uzar U. Examining the roles of renewable energy consumption and agriculture on CO2 emission in lucky-seven countries. Environ Sci Pollut Res Int. 2020;27(36):45031–40. doi: 10.1007/s11356-020-10374-2 32772294 PMC7477878

[19] Zhou G, Li H, Ozturk I, Ullah S. Shocks in agricultural productivity and CO2 emissions: new environmental challenges for China in the green economy. Economic Research-Ekonomska Istraživanja. 2022:1–17.

[20] Rahman MM, Nepal R, Alam K. Impacts of human capital, exports, economic growth and energy consumption on CO2 emissions of a cross-sectionally dependent panel: Evidence from the newly industrialized countries (NICs). Environmental Science & Policy. 2021;121:24–36. doi: 10.1016/j.envsci.2021.03.017

[21] Zaman Q uz, Wang Z, Zaman S, Rasool SF. Investigating the nexus between education expenditure, female employers, renewable energy consumption and CO2 emission: Evidence from China. Journal of Cleaner Production. 2021;312:127824. doi: 10.1016/j.jclepro.2021.127824

[22] Mentel U, Wolanin E, Eshov M, Salahodjaev R. Industrialization and CO2 Emissions in Sub-Saharan Africa: The Mitigating Role of Renewable Electricity. Energies. 2022;15(3):946. doi: 10.3390/en15030946

[23] Zhou C, Wang S, Feng K. Examining the socioeconomic determinants of CO2 emissions in China: A historical and prospective analysis. Resources, Conservation and Recycling. 2018;130:1–11. doi: 10.1016/j.resconrec.2017.11.007

[24] Yin Y, Xiong X, Ullah S, Sohail S. Examining the asymmetric socioeconomic determinants of CO2 emissions in China: challenges and policy implications. Environ Sci Pollut Res Int. 2021;28(40):57115–25. doi: 10.1007/s11356-021-14608-9 34081281

[25] Zheng H, Hu J, Guan R, Wang S. Examining Determinants of CO2 Emissions in 73 Cities in China. Sustainability. 2016;8(12):1296. doi: 10.3390/su8121296

[26] Azam M, Uddin I, Khan S, Tariq M. Are globalization, urbanization, and energy consumption cause carbon emissions in SAARC region? New evidence from CS-ARDL approach. Environ Sci Pollut Res Int. 2022;29(58):87746–63. doi: 10.1007/s11356-022-21835-1 35821313

[27] Voumik LC, Mimi MB, Raihan A. Nexus Between Urbanization, Industrialization, Natural Resources Rent, and Anthropogenic Carbon Emissions in South Asia: CS-ARDL Approach. Anthr Sci. 2023;2(1):48–61. doi: 10.1007/s44177-023-00047-3

[28] Cui X, Wang W, Işık C, Uddin I, Yan J, Gu X, et al. Do geopolitical risk and economic policy uncertainty cause CO2 emissions in BRICS? The role of institutional quality and energy productivity. Stoch Environ Res Risk Assess. 2024;38(5):1685–99. doi: 10.1007/s00477-023-02646-3

[29] Du J, Ahmad M, Uddin I, Xu H, Gu X. From growth to sustainability: investigating N-shaped EKC and the role of energy productivity, technological advancement, and human capital in OECD economies. Environ Sci Pollut Res Int. 2023;30(46):102374–88. doi: 10.1007/s11356-023-29514-5 37667124

[30] Liu H, Evans S, Zhang Z, Song W, You X. The Carbon Brief profile: China. Carbon Brief. 2023. Retrieved from https://interactive.carbonbrief.org/the-carbon-brief-profile-china/index.html

[31] Zhou X, Liu Z, Wu L, Wang Y. Study on CO2 Emission Forecast of “Four Provinces of Mountains and Rivers” Based on Time-SeriesMachine Learning. Atmosphere. 2024;15(8):949. doi: 10.3390/atmos15080949

[32] Uddin I, Usman M, Saqib N, Makhdum MSA. The impact of geopolitical risk, governance, technological innovations, energy use, and foreign direct investment on CO2 emissions in the BRICS region. Environ Sci Pollut Res Int. 2023;30(29):73714–29. doi: 10.1007/s11356-023-27466-4 37195610

[33] Misra AK, Verma M. Impact of environmental education on mitigation of carbon dioxide emissions: a modelling study. IJGW. 2015;7(4):466. doi: 10.1504/ijgw.2015.070046

[34] Alkhateeb TTY, Mahmood H, Altamimi NN, Furqan M. Role of education and economic growth on the CO2 emissions in Saudi Arabia. Entrepreneurship and Sustainability Issues. 2020;8(2):195.

[35] Eyuboglu K, Uzar U. A new perspective to environmental degradation: the linkages between higher education and CO2 emissions. Environ Sci Pollut Res Int. 2021;28(1):482–93. doi: 10.1007/s11356-020-09414-8 32815009

[36] Lee H, Park C, Jung H. The role of tertiary education on CO2 emissions: evidence from 151 countries. Environment, Development and Sustainability. 2024:1–23.

[37] Sahu M, Prusty T, Alahdal WM, Ariff AM, Almaqtari FA, Hashim HA. The role of education in moderating the impact of development on environmental sustainability in OECD countries. Discov Sustain. 2024;5(1):237. doi: 10.1007/s43621-024-00450-9

[38] Li H, Khattak SI, Ahmad M. Measuring the impact of higher education on environmental pollution: new evidence from thirty provinces in China. Environ Ecol Stat. 2021;28(1):187–217. doi: 10.1007/s10651-020-00480-2

[39] Zhu T-T, Peng H-R, Zhang Y-J, Liu J-Y. Does higher education development facilitate carbon emissions reduction in China. Applied Economics. 2021;53(47):5490–502. doi: 10.1080/00036846.2021.1923641

[40] Cui Y, Wei Z, Xue Q, Sohail S. Educational attainment and environmental Kuznets curve in China: an aggregate and disaggregate analysis. Environ Sci Pollut Res Int. 2022;29(30):45612–22. doi: 10.1007/s11356-022-19051-y 35147882

[41] Lin Y-N, Chiu Y-H, Chang T-H, Lin T-Y, Chiu S-Y. The impact of education level on residents’ carbon consumption in China. Int J Environ Sci Technol. 2022;20(9):9603–18. doi: 10.1007/s13762-022-04626-6

[42] Dong Y, Gao J, Qiu J, Cui Y, Guo M. The dynamic spatial effects of education investment on carbon emissions: heterogeneous analysis based on north-south differences in China. Front Environ Sci. 2024;12:1432457. doi: 10.3389/fenvs.2024.1432457

[43] Zhao N, Wang C, Shi C, Liu X. The effect of education expenditure on air pollution: Evidence from China. J Environ Manage. 2024;359 121006. doi: 10.1016/j.jenvman.2024.121006 38692028

[44] Ekperiware MC, Olatayo TO, Egbetokun AA. Human capital and sustainable development in Nigeria: How can economic growth suffice environmental degradation? (No. 2017-29). Economics Discussion Papers. 2017.

[45] Mukherjee S. A study of awareness towards environmental degradation among the urban and rural undergraduate students. Aensi. In. 2018.

[46] Dietz T, Rosa EA. Rethinking the environmental impacts of population, affluence and technology. Human ecology review. 1994;1(2):277–300.

[47] Ehrlich PR, Holdren JP. Impact of Population Growth: Complacency concerning this component of man's predicament is unjustified and counterproductive. Science. 1971;171(3977):1212–7. doi: 10.1126/science.171.3977.1212 5545198

[48] York R, Rosa EA, Dietz T. STIRPAT, IPAT and ImPACT: analytic tools for unpacking the driving forces of environmental impacts. Ecological Economics. 2003;46(3):351–65. doi: 10.1016/s0921-8009(03)00188-5

[49] Anser MK, Syed QR, Apergis N. Does geopolitical risk escalate CO2 emissions? Evidence from the BRICS countries. Environ Sci Pollut Res Int. 2021;28(35):48011–21. doi: 10.1007/s11356-021-14032-z 33900560

[50] Dickey DA, Fuller WA. Distribution of the Estimators for Autoregressive Time Series With a Unit Root. Journal of the American Statistical Association. 1979;74(366):427. doi: 10.2307/2286348

[51] Phillips PCB, Perron P. Testing for a unit root in time series regression. Biometrika. 1988;75(2):335–46. doi: 10.1093/biomet/75.2.335

[52] Gujarati DN. Basic econometrics. 2009.

[53] Johansen S, Juselius K. Maximum likelihood estimation and inference on cointegration — with applications to the demand for money. Oxf Bull Econ Stat. 1990;52(2):169–210. doi: 10.1111/j.1468-0084.1990.mp52002003.x

[54] Pedroni P. Fully modified OLS for heterogeneous cointegrated panels. In: Nonstationary panels, panel cointegration, and dynamic panels. Emerald Group Publishing Limited; 2001:93–130.

[55] Khan MWA, Panigrahi SK, Almuniri KSN, Soomro MI, Mirjat NH, Alqaydi ES. Investigating the Dynamic Impact of CO2 Emissions and Economic Growth on Renewable Energy Production: Evidence from FMOLS and DOLS Tests. Processes. 2019;7(8):496. doi: 10.3390/pr7080496

[56] Ivascu L, Sarfraz M, Mohsin M, Naseem S, Ozturk I. The Causes of Occupational Accidents and Injuries in Romanian Firms: An Application of the Johansen Cointegration and Granger Causality Test. Int J Environ Res Public Health. 2021;18(14):7634. doi: 10.3390/ijerph18147634 34300085 PMC8307420

[57] Khan MA, Uddin I, Othman NS. Factors determining water stress: do environmental distress, energy consumption and industrialization matter?. Environment, Development and Sustainability. 2024:1–25.

[58] Ochi A, Saidi A. Impact of governance quality, population and economic growth on greenhouse gas emissions: An analysis based on a panel VAR model. J Environ Manage. 2024;370:122613. doi: 10.1016/j.jenvman.2024.122613 39326084

[59] Misra AK, VERMA M. Impact of industrialization on the dynamics of atmospheric carbon dioxide: a modeling study. International Journal of Big Data Mining for Global Warming. 2022;4(1):2150009.

[60] Mehmood S, Zaman K, Khan S, Ali Z, Khan Hur R. The role of green industrial transformation in mitigating carbon emissions: Exploring the channels of technological innovation and environmental regulation. Energy and Built Environment. 2024;5(3):464–79. doi: 10.1016/j.enbenv.2023.03.001

[61] Long X, Chen Y, Du J, Oh K, Han I. Environmental innovation and its impact on economic and environmental performance: Evidence from Korean-owned firms in China. Energy Policy. 2017;107:131–7. doi: 10.1016/j.enpol.2017.04.044

[62] Wang R, Mirza N, Vasbieva DG, Abbas Q, Xiong D. The nexus of carbon emissions, financial development, renewable energy consumption, and technological innovation: What should be the priorities in light of COP 21 Agreements? J Environ Manage. 2020;271:111027. doi: 10.1016/j.jenvman.2020.111027 32778307

[63] Khan F, Rehman ZU. Empowering women is an approach to development of a nation: A case study of Pakistan. VFAST Transactions on Education and Social Sciences. 2021;9(2):12–8.

