# Supplementary material for: Revisiting the determinants of CO2 emissions: The role of higher education under the extended STIRPAT model
Source: PLoS One. 2025 Mar 18;20(3):e0319930. doi: 10.1371/journal.pone.0319930 (PMC11919276; doi:10.1371/journal.pone.0319930)
Supplement: S1 Table — (PDF) [file pone.0319930.s001.pdf]

Series Name	GDP per capita (constant 2015 US$)	Carbon dioxide (CO2) emissions (total) excluding LULUCF (Mt CO2e)	Patent applications, nonresidents	Patent applications, residents	Technological Innovation	Population, total	Industry (including construction), value added (constant 2015 US$)	School enrollment, tertiary (% gross)
1985	667.1285	1871.064	4493	4065	8558	1.05E+09	1.9E+11	2.19178
1986	716.1053	1987.003	4515	3494	8009	1.07E+09	2.09E+11	2.77602
1987	786.8649	2126.001	4084	3975	8059	1.08E+09	2.38E+11	3.04914
1988	861.1935	2285.186	4872	4780	9652	1.1E+09	2.72E+11	3.08643
1989	883.7642	2364.078	4910	4749	9659	1.12E+09	2.82E+11	3.01468
1990	905.0325	2406.18	4305	5832	10137	1.14E+09	2.91E+11	3.00149
1991	975.4629	2542.074	4051	7372	11423	1.15E+09	3.31E+11	2.88531
1992	1100.646	2661.706	4387	10022	14409	1.16E+09	4E+11	2.81975
1993	1239.129	2882.766	7534	12084	19618	1.18E+09	4.8E+11	2.9364
1994	1384.93	3039.54	7876	11191	19067	1.19E+09	5.67E+11	3.77982
1995	1520.029	3360.563	8688	10011	18699	1.2E+09	6.45E+11	4.67359
1996	1653.434	3334.631	11114	11628	22742	1.22E+09	7.23E+11	5.29578
1997	1787.767	3430.838	12102	12672	24774	1.23E+09	7.99E+11	5.82305
1998	1909.622	3515.385	33645	13751	47396	1.24E+09	8.7E+11	6.28842
1999	2038.206	3441.209	34418	15626	50044	1.25E+09	9.41E+11	6.62029
2000	2193.897	3659.95	26560	25346	51906	1.26E+09	1.03E+12	7.5585
2001	2359.572	3837.991	33412	30038	63450	1.27E+09	1.12E+12	9.22886
2002	2557.892	4143.91	40426	39806	80232	1.28E+09	1.23E+12	11.71571
2003	2797.177	4785.013	48548	56769	105317	1.29E+09	1.38E+12	14.39121
2004	3061.833	5515.057	64598	65786	130384	1.3E+09	1.54E+12	16.77984

2005	3390.716	6258.413	79842	93485	173327	1.3E+09	1.72E+12	18.55515
2006	3800.766	6944.311	88183	122318	210501	1.31E+09	1.96E+12	20.50988
2007	4319.031	7547.621	92101	153060	245161	1.32E+09	2.25E+12	21.44643
2008	4711.643	7774.955	95259	194579	289838	1.32E+09	2.47E+12	21.85469
2009	5128.904	8340.201	85508	229096	314604	1.33E+09	2.73E+12	23.27021
2010	5647.069	9124.911	98111	293066	391177	1.34E+09	3.07E+12	25.28607
2011	6152.697	9975.931	110583	415829	526412	1.35E+09	3.4E+12	26.80753
2012	6591.662	10288.86	117464	535313	652777	1.35E+09	3.69E+12	29.32504
2013	7056.423	10782.99	120200	704936	825136	1.36E+09	3.98E+12	32.76203
2014	7532.785	10909.72	127042	801135	928177	1.37E+09	4.26E+12	43.88219
2015	8016.446	10768.98	133612	968252	1101864	1.38E+09	4.52E+12	47.43538
2016	8516.529	10786.1	133522	1204981	1338503	1.39E+09	4.79E+12	49.70836
2017	9053.229	11026.11	135885	1245709	1381594	1.4E+09	5.07E+12	51.58277
2018	9619.209	11553.54	148187	1393815	1542002	1.4E+09	5.37E+12	54.00762
2019	10155.51	11819.36	157093	1243568	1400661	1.41E+09	5.63E+12	57.27544
2020	10358.17	12037.32	152342	1344817	1497159	1.41E+09	5.77E+12	62.23678
2021	11223.26	12717.66	159019	1426644	1585663	1.41E+09	6.27E+12	67.38762
2022	11560.24	12667.43	156151.3	1338343	1494494	1.41E+09	6.5E+12	71.98155
2023	12174	12474.13	155837.4	1369935	1525772	1.41E+09	6.73E+12	74.8236
